# Ganfule capsule alleviates bile duct ligation-induced liver fibrosis in mice by inhibiting glutamine metabolism

**DOI:** 10.3389/fphar.2022.930785

**Published:** 2022-10-07

**Authors:** Chang Ke, Jianlong Gao, Jiyuan Tu, Yan Wang, Yangxin Xiao, Yuan Wu, Yanju Liu, Zhongshi Zhou

**Affiliations:** ^1^ College of Pharmacy, Hubei University of Chinese Medicine, Wuhan, Hubei, China; ^2^ Department of Minimally Invasive Interventional Oncology, Hubei Cancer Hospital, Tongji Medical College, Huazhong University of Science and Technology, Wuhan, Hubei, China; ^3^ Center for Hubei TCM Processing Technology Engineering, Wuhan, Hubei, China; ^4^ Department of Radiation Oncology, Hubei Cancer Hospital, Tongji Medical College, Huazhong University of Science and Technology, Wuhan, Hubei, China

**Keywords:** ganfule capsule, bile duct ligation, liver fibrosis, glutamine metabolism, NF-κB pathway

## Abstract

**Background**: Liver fibrosis is a pathological outcome of a variety of liver diseases, and it can also progress into liver cirrhosis and liver cancer. Specific liver antifibrotic drugs have not been clinically approved yet. Studies have demonstrated the protective effects of Ganfule capsule (GFL) on the liver and its therapeutic potential in hepatic cancer. However, the mechanism of GFL is not clear in the treatment of liver fibrosis.

**Objective**: This article aims to study the protective effect of GFL on liver fibrosis and its possible mechanism.

**Methods:** The cholestatic liver fibrosis model was prepared by subjecting C57BL/6 mice to bile duct ligation (BDL). The GFL groups were treated with different concentrations of GFL for 14 days. Pathological analysis, serum biochemical index detection, metabonomic analysis, immunohistochemistry, Western blot, and real-time PCR were carried out.

**Results**: GFL could alleviate liver injury and liver fibrosis caused by BDL in mice. Metabonomic analysis of mice serum showed postoperative metabolic disorder, which could be alleviated by GFL through glutamine metabolism; valine, leucine, and isoleucine biosynthesis; aminoacyl-tRNA biosynthesis; and other metabolic pathways. GFL affected glutamine metabolism by inhibiting the activity of glutaminase 1 (GLS1). The activation of GLS1 is regulated by the NF-κB pathway, and experiments showed that GFL could inhibit IκB-α and NF-κB p65 phosphorylation.

**Conclusion:** This study confirms the protective effect of GFL on liver injury and shows that GFL inhibits glutamine metabolism, which was correlated with the NF-κB pathway, and eventually alleviates liver fibrosis. These results are conducive to the development of new therapeutic drugs for liver fibrosis.

## 1 Introduction

Liver fibrosis is a response initiated against the damage to the liver that is mainly caused by continuous and extensive liver inflammation. The development of progressive liver fibrosis is associated with multiple etiologies, including chronic viral infection, nonalcoholic fatty liver disease, nonalcoholic steatohepatitis, alcoholism, autoimmune hepatitis, and biliary tract disease (Kisseleva and Brenner, 2021; Pan et al., 2021). These liver diseases induce chronic liver injury that consequently promotes the persistent activation of hepatic stellate cells (HSCs). As a result, extracellular matrix (ECM) proteins accumulate and disrupt the structure and the function of the liver (Henderson et al., 2020). If untreated, liver fibrosis can progress to cirrhosis and hepatocellular carcinoma. Moreover, it is the leading cause of liver-related global morbidity and mortality (Kisseleva and Brenner, 2021). Although the potential of some drugs to provide a multidimensional protective effect against the progression of liver fibrosis is under investigation in clinical trials (Loomba et al., 2018; Harrison et al., 2020), no specific drug against liver fibrosis has been approved for clinical use so far. The only curative treatment option available for patients with advanced liver cirrhosis is liver transplantation (Neong et al., 2019). Therefore, it is imperative to accelerate the development of drugs for liver fibrosis.

The treatment of liver fibrosis may involve multiple targets and multiple pathways, and the regulation of multiple targets is the advantage of traditional Chinese medicine (TCM). The great potential of TCM to treat liver fibrosis has been proved in previous studies (Duval et al., 2015; Latief and Ahmad, 2018). The Ganfule capsule (GFL) is a typical TCM, approved by the National Medical Products Administration of China, for the clinical treatment of hepatocellular carcinoma. Plenty of clinical data and experimental studies have proved the good therapeutic effect of GFL on hepatocellular carcinoma (Gao, 2014; Zhang et al., 2021). In addition, clinical research has highlighted the efficacy of GFL in treating biliary cirrhosis, liver fibrosis, and hepatitis (Huang and D Tong, 2022; M Wang and T Liu, 2016; Xun et al., 2020). However, the possible mechanism of GFL in the treatment of liver fibrosis is not known.

As the liver is the metabolic center of the body, a liver injury will inevitably elicit abnormal metabolism, which will lead to the accumulation of a large number of fibroblasts followed by the aggravating process of liver fibrosis (Schwabe et al., 2020). A series of studies have shown that the key enzymes of glutaminolysis are significantly elevated in fibrosis that subsequently accelerate the conversion of glutamine to glutamate and eventually confer HSC resistance to apoptosis (Bai et al., 2019). Inhibiting the key enzyme glutaminase 1 (GLS1) for glutamine decomposition can inhibit live fibrosis and alleviate liver damage (Trivedi et al., 2021). Furthermore, other metabolic pathways may also serve as important drivers of fibroblast activation, such as increased glucose metabolism and enhanced fatty acid oxidation (Wallace et al., 2015; Nigdelioglu et al., 2016; Park et al., 2018; Liu and Summer 2019). Previous studies have demonstrated that GFL can regulate metabolic pathways of amino acids, lipids, and carbohydrates to help cure liver cancer (Xu et al., 2022). Thus, it is worth exploring whether GFL alleviates liver fibrosis by regulating metabolism.

In this study, we determined the pharmacological effects of GFL to relieve the BDL-induced liver injury and the potential of GFL to inhibit liver fibrosis. Furthermore, the metabolic regulation effect of GFL on mice with BDL was also proved, particularly in terms of glutamine metabolism. This may be achieved by regulating GLS1 activity through the NF-κB pathway.

## 2 Materials and methods

### 2.1 Chemicals and reagents

Ganfule capsule was purchased from Changsha Kamp Medicine Co., Ltd. (Changsha, China); a total of 21 ingredients were used, including Codonopsis Radix, Trionycis Carapax, Paridis Rhizoma, Atractylodis Macrocephalae Rhizoma, and Astragali Radix. The total drug name, family, and scientific name are shown in Table 1; methanol (cat. #67-56-1) was purchased from Merck (Darmstadt, Germany), and acetonitrile (cat. #51101) was purchased from Thermo Fisher Scientific Inc. (Shanghai China). N-methyl-N-trimethylsilyl-trifluoro-acetamide (BSTFA, #FM05241802), methoxyamine hydrochloride (#BCBZ8981), and pyridine (#SHBK6453) were obtained from Sigma Aldrich, Co., St. (St. Louis, MO, USA).

**TABLE 1:** Composition of GFL capsules.

No.	Drug name	Family name	Scientific names
1	Codonopsis Radix	Campanulaceae	*Codonopsis pilosula* (*Franch.*) *Nannf*
2	Trionycis Carapax	Trionylidae	*Trionyx sinensis Weigmann*
3	Paridis Rhizoma	Liliaceae	*Paris polyphylla var. chinensis (Franch.) H.Hara*
4	Atractylodis Macrocephalae Rhizoma	Asteraceae	*Atractylodes macrocephala Koidz*
5	Astragali Radix	Fabaceae	*Astragalus mongholicus Bunge*
6	Citri Reticulatae Pericarpium	Rutaceae	*Citrus reticulata Blanco*
7	Eupolyphage Steleophaga	Cockroachidae	*Steleophaga plancyi(Boleny)*
8	Rhei Radix et Rhizoma	Polygonaceae	*Rheum palmatum L*
9	Persicae Semen	Rosaceae	*Prunus persica*(*L.*)*Batsch*
10	Scutellariae Barbatae Herba	Lamiaceae	*Scutellaria barbata D.Don*
11	Patriniae Herba	Caprifoliaceae	*Patrinia villosa (Thunb.) Dufr*
12	Poria	Polyporaceae	*Poria cocos (Schw.) Wolf *
13	Coicis Semen	Poaceae	*Coix lacryma-jobi L. var. ma-yuen* (*Roman.*)*Stapf*
14	Curcumae Radix	Zingiberaceae	*Curcuma longaL*
15	Sappan Lignum	Fabaceae	*Biancaea sappan (L.) Tod*
16	Ostreae Concha	Ostreidae	*Ostrea gigas Thunberg*
17	Artemisiae Scopariae Herba	Asteraceae	*Artemisia scoparia Waldst. et Kit*
18	Akebiae Caulis	Lardizabalaceae	*Akebia quinata*(*Thunb.*)*Decne*
19	Cyperi Rhizoma	Cyperaceae	*Cyperus rotundus L*
20	Aquilariae Lignum Resinatum	Thymelaeaceae	*Aquilaria sinensis*(*Lour.*)*Gilg*
21	Bupleuri Radix	Apiaceae	*Bupleurum chinense DC.*

ABScript III RT SuperMix (RK20429; ABclonal Technology, Wuhan, China) and universal SYBR qPCR Master Mix (Q711-02; Vazyme Biotech Co., Ltd., Nanjing, China) were used.

Glutamic-Pyruvic Transaminase (ALT) Assay Kit (cat. # BC1555), Glutamic-Oxalacetic Transaminase (AST) Assay Kit (cat. # BC1565), and Glutamic Acid (Glu) Content Assay Kit (cat. #BC1585) were purchased from Solarbio (Beijing, China). Total cholesterol (TC) colorimetric Assay Kit (cat. #E-BC-K109-M), triglyceride (TG) colorimetric Assay Kit (cat. #E-BC-K261-M), Total Bilirubin (TBIL) Colorimetric Assay Kit (cat. #E-BC-K760-M), mouse interleukin 1 Beta (IL-1β) ELISA Kit (cat. #E-EL-M0037c), and mouse tumor necrosis factor alpha (TNF-α) ELISA Kit (cat. #E-EL-M3063) were purchased from Elabscience Biotechnology Co., Ltd. (Wuhan, China). Antibodies against GAPDH (60004-1-Ig), α-SMA (14395-1-AP), and COL1A1 (67288-1-Ig) were purchased from Proteintech (Wuhan, China). Antibodies against GLS1 (A3885) and GLS2 (A16029) were purchased from ABclonal Technology (Wuhan, China). Antibodies against IκBα (#9242), phospho-IκBα (#5209), NF-κB p65 (#8242), and phospho-NF-κB p65 (#3033) were purchased from CST (Boston, MA, United States).

### 2.2 Preparation of GFL

According to the description of the instruction manual, the daily intake of GFL for adult is 9 g, and the dose for mice is calculated according to the body surface area: 9 g × 0.0026/0.02 kg = 1.17 g/kg. After grinding and crushing, the contents of GFL were dissolved in 0.9% NaCl solution and were processed for ultrasonic oscillation to prepare suspensions. In total, two strengths of suspensions were obtained, namely, 0.117 g/ml and 0.234 g/ml, which represent the low dose (1.17 g/kg) and high dose (2.34 g/kg) of GFL, respectively. The prepared GFL was stored at −20°C in dark.

### 2.3 LC-MS analysis

The GFL was accurately weighed (200 mg ± 1%) in a 2-ml Eppendorf tube, and 0.6 ml 2-chlorophenyl alanine (4 ppm) methanol (−20°C) was added. The contents were ground in the tissue grinder for 90 s at 60 Hz. Following the centrifugation at 12,000 rpm at 4°C for 10 min, the supernatant was collected with a 0.22-μm microporous membrane for filtration, and the filtered solution was added to a detection bottle.

Chromatographic separation was used with an ACQUITY UPLC® HSS T3 (150 × 2.1 mm, 1.8 µm, Waters), and the column was maintained at 40°C. The temperature of the autosampler was 8°C. Gradient elution of analytes was carried out with 0.1% formic acid in water and 0.1% formic acid in acetonitrile or 5 mM ammonium formate in water and acetonitrile at a flow rate of 0.25 ml/min. A volume of 2 μl of each sample was injected after equilibration. An increasing linear gradient of solvent B (v/v) was used as follows: 0–1 min, 2% B/D; 1–9 min, 2%–50% B/D; 9–12 min, 50%–98% B/D; 12–13.5 min, 98% B/D; 13.5–14 min, 98%–2% B/D; and 14–20 min, 2% D positive model (14–17 min, 2% B-negative model). The ESI-MSn experiments were performed with a spray voltage of 3.5 kV and −2.5 kV in positive and negative modes, respectively. Sheath gas and auxiliary gas were set at 30 and 10 arbitrary units, respectively. The capillary temperature was 325°C. The Orbitrap analyzer scanned over a mass range of m/z 81-1 000 for a full scan at a mass resolution of 70,000. Data-dependent acquisition LC-MS experiments were performed with an HCD scan. The normalized collision energy was 30 eV. Dynamic exclusion was implemented to remove some unnecessary information in MS/MS spectra.

### 2.4 Animals

C57/BL6 male mice aged 6–7 weeks and with a bodyweight of about 20 ± 2 g were provided by Liaoning Changsheng Biotechnology Co., Ltd., Certificate No. SCXK (L) 2020-0001. The mice were bred for adaptation for 3 days under laboratory conditions at a temperature of 23 ± 2°C, humidity of 55 ± 5%, and a 12-h light/dark cycle, with food and water *ad libitum*. All the animal experiments were approved by the Animal Ethics Committee of the Hubei University of Chinese Medicine.

### 2.5 Model establishment and grouping

A total of 48 male C57/BL6 mice were randomly assigned to four groups: sham-operated, model, GFL-LD (1.17 g/kg), and GFL-HD (2.34 g/kg). All animals were anesthetized with sodium pentobarbital. The abdominal cavity of all groups was opened by using sterilized surgical instruments. After that, the bile duct was separated, ligated, and finally sutured in all groups, except the sham-operated group in which the abdominal cavity was directly sutured without ligation. Following 24 h of operation, sham and model groups were gavaged with 0.1 ml/10 g of 0.9% NaCl, whereas GFL-LD and GFL-HD groups were gavaged using 0.1 ml/10 g of 0.117 g/ml and 0.234 g/ml GFL suspensions, respectively. All animals were euthanized after 14 days of treatment (Dold et al., 2009; Ge et al., 2017).

### 2.6 Histopathology

The livers of sacrificed animals were fixed in a 4% neutral formaldehyde solution for 24 h, dehydrated in ethanol, embedded in paraffin, and were cut into 3–4 μM slices. The slices were dewaxed with xylene, rewatered with gradient ethanol, stained with hematoxylin and eosin, washed in ethanol, and clarified and encapsulated in xylene (Qu et al., 2022a).

### 2.7 Sirius red staining

The slices were sequentially dewaxed with xylene, rewatered with gradient ethanol, stained with Celestine Blue solution and Picro Sirius Red, washed in ethanol, and finally clarified and encapsulated in xylene.

### 2.8 Masson staining

The slices were dewaxed with xylene, rewatered with gradient ethanol, and stained with hematoxylin and Masson complex staining solution. The slices were then differentiated using 1% phosphomolybdic acid aqueous, stained with aniline blue, washed in ethanol, and clarified and encapsulated in xylene.

### 2.9 Immunohistochemistry

The liver tissue was first fixed by immersion in 4% paraformaldehyde for 24 h. The fixed liver tissue was then dehydrated in ethanol and embedded in paraffin. Next, 3–4 μM thick sections of the tissue were obtained and incubated with primary antibody overnight. The tissue sections were subsequently washed three times with phosphate-buffered solution and incubated with secondary antibody at 37°C for 30 min. The positive cells were shown in brown.

### 2.10 RNA isolation and RT-qPCR

Total RNA was extracted employing the TRIzol reagent and was reverse transcribed into cDNA using a reverse transcription kit with a genomic DNA scavenger under RNase-free conditions. The primer sequences are shown in Supplementary Table S1. Melt curves were analyzed to verify the specificity of PCR products and SYBR-based quantitative PCR was exploited to measure gene amplification. The order, number, and temperature of PCR cycles were as follows: 40 cycles at 95°C for 1 min, 40 cycles at 95°C for 20 s, 40 cycles at 60°C for 45 s, and 40 cycles at 95°C for 1 min. β-actin was used as the endogenous control for normalization (Qu et al., 2022b).

### 2.11 Western blotting

Liver tissue was processed for lysis using radioimmunoprecipitation assay (RIPA) buffer containing total protease inhibitor and phosphatase inhibitor. Total protein was isolated by using the sodium dodecyl sulfate–polyacrylamide gel electrophoresis (SDS-PAGE) technique and transferred to polyvinylidene difluoride membranes followed by sealing with 5.0% skimmed milk powder at room temperature for 2 h. The membranes were incubated with primary antibodies on a shaker at −4°C for 12 h. After washing, incubation was performed with secondary antibodies (1:5000) at room temperature for 1 h. An ECL chemiluminescence detection kit was utilized to visualize protein bands. The quantity of each protein was estimated by reference to a GAPDH standard.

### 2.12 Preparation of serum samples

Serum samples, which were refrigerated at −80°C, were taken out, thawed at 4°C, and centrifuged at 3,000 rpm at 4°C for 15 min. Next, 50 µl of the supernatant was transferred into a 1.5-ml EP tube in which 150 µl methanol was added, and the resulting mixture was vortexed and centrifuged at 12,000 rpm for 10 min at 4°C. A volume of 185 µl of the supernatant was collected into a 1.5-ml EP tube and dried under a stream of nitrogen. A volume of 80 µl of methoxy-pyridine solution (20 mg/ml) was added to the dried supernatant, and the mixture was processed for vortexing, centrifugation, and incubation in a water bath at 80°C for 15 min. After adding 80 µl of BSTFA, the sample was vortexed, centrifuged, and incubated at 80°C in a water bath for 15 min. The samples were then cooled for 5 min, vortexed for 15 s, and centrifuged at 12,000 rpm for 10 min at 4°C. Finally, 100 µl supernatant was collected in the injection bottle with an inner liner. The samples were stored at 4°C and were analyzed by GC-MS within 24 h after preparation (Linghang et al., 2020). The quality control (QC) sample was prepared by evenly mixing 4 μl of solution from each injection bottle of samples and transferring to a new injection bottle.

### 2.13 GC-MS analysis

Samples in a quantity of 1 µl each were injected into the gas chromatograph system comprising a split inlet equipped with a DB-5MS capillary column (30.0 µm × 250 µm inner diameter, 0.25 µm film thickness) under the following conditions: The oven temperature was initially maintained at 80°C for 3 min, and then it was increased to 140°C at a rate of 7°C/min for 4 min, to 180°C at a rate of 5°C/min for 6 min, and to 280°C at a rate of 5°C/min for 2 min. Helium was used as the carrier gas at a constant flow rate of 1 ml/min with an injector split ratio set to 10:1. The temperatures of the injector and MS were set to 280°C, whereas the temperature of the ion source was set to 200°C. The energy was set to −70 eV in the electron ionization mode. MS data were acquired in the full-scan mode across a mass-to-charge ratio (m/z) range of 50–650 (Pan et al., 2022).

### 2.14 Metabonomic analysis

The metabonomic peak identification, alignment, and filtering were performed using XCMS Online, and it resulted in a data matrix consisting of m/z values, retention times, ion fragments, and peak areas. GC-MS data files of all serum samples were further converted to the CSV format and imported into SIMCA-P14.1 software (Umetrics AB, Umea, Sweden) for normalization and a multivariate statistical analysis. PCA, OPSL DA, and other analysis were performed in SIMCA-P14.1. To ascertain the overall contribution of the corresponding metabolites in the OPLS-DA model, the variable importance in projection (VIP) score of each metabolite peak was calculated. Compounds with VIP < 1 and *p* < 0.05 were considered as differential metabolites. Differential metabolites were identified using the GC-MS database. Metaboanalyst (Pang et al., 2021) was used for enrichment analysis and pathway analysis of differential metabolites (Li et al., 2022).

### 2.15 Cell culture

LX-2 cells were mixed with 10% fetal bovine serum, 100 U/mL of streptomycin, 100 U/mL of penicillin, and DMEM medium and incubated at 37°C and 5% CO_2_. When the number of cells was sufficient, a part of the cells was retrieved and frozen. A cryopreservation solution comprises 10% DMSO, 40% fetal bovine serum, and 50% DMEM culture medium. After determining the toxicity of GFL by using the CCK-8 assay, LX-2 cells were seeded in dishes for 24 h (Feng et al., 2021). Then, the fluid was changed only in the control group, and the model group was administered with 5 ng/ml of TGF-β1. The groups of GFL (250 μg/ml) and GFL (500 μg/ml) were given 5 ng/ml of TGF-β1 and corresponding doses of drugs. Cells were collected for detection after 24 h.

### 2.16 Statistical analysis

All the statistical analyses were performed using ANOVA in GraphPad Prism version 8.0 software (San Diego, CA, USA). The results were expressed as means ± standard deviation. Differences were considered statistically significant at *p* < 0.05.

## 3 Results

### 3.1 The chemical components of the GFL

**FIGURE 1:**
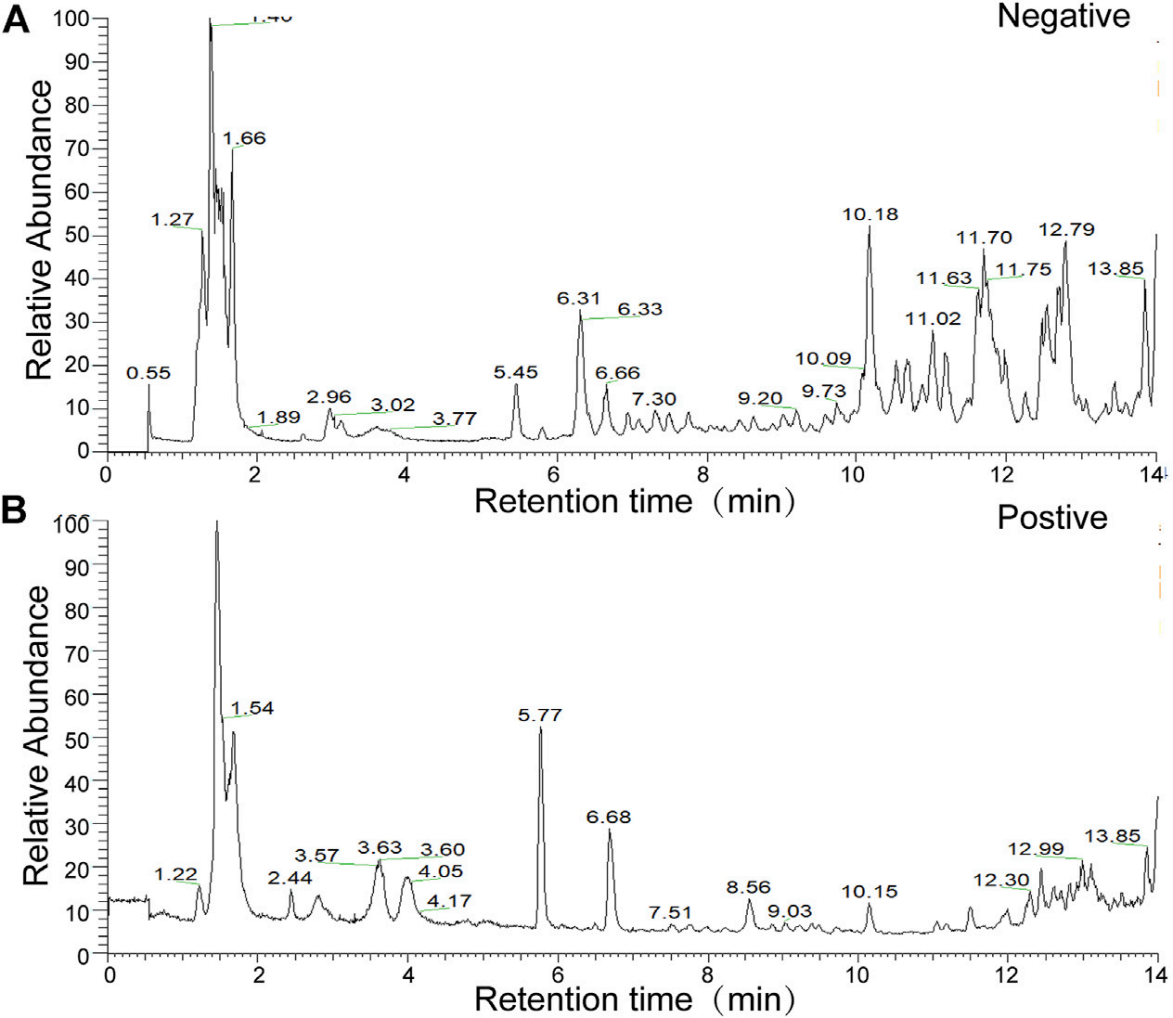
Total ion flow chart of sample in the **(A)** negative-ion mode **(B)** and in the positive-ion mode.

The present study identified 32 compounds in GFL through LC-MS (Figure 1 and Supplementary Table S2).

### 3.2 GFL inhibits liver injury and inflammation caused by BDL

**FIGURE 2:**
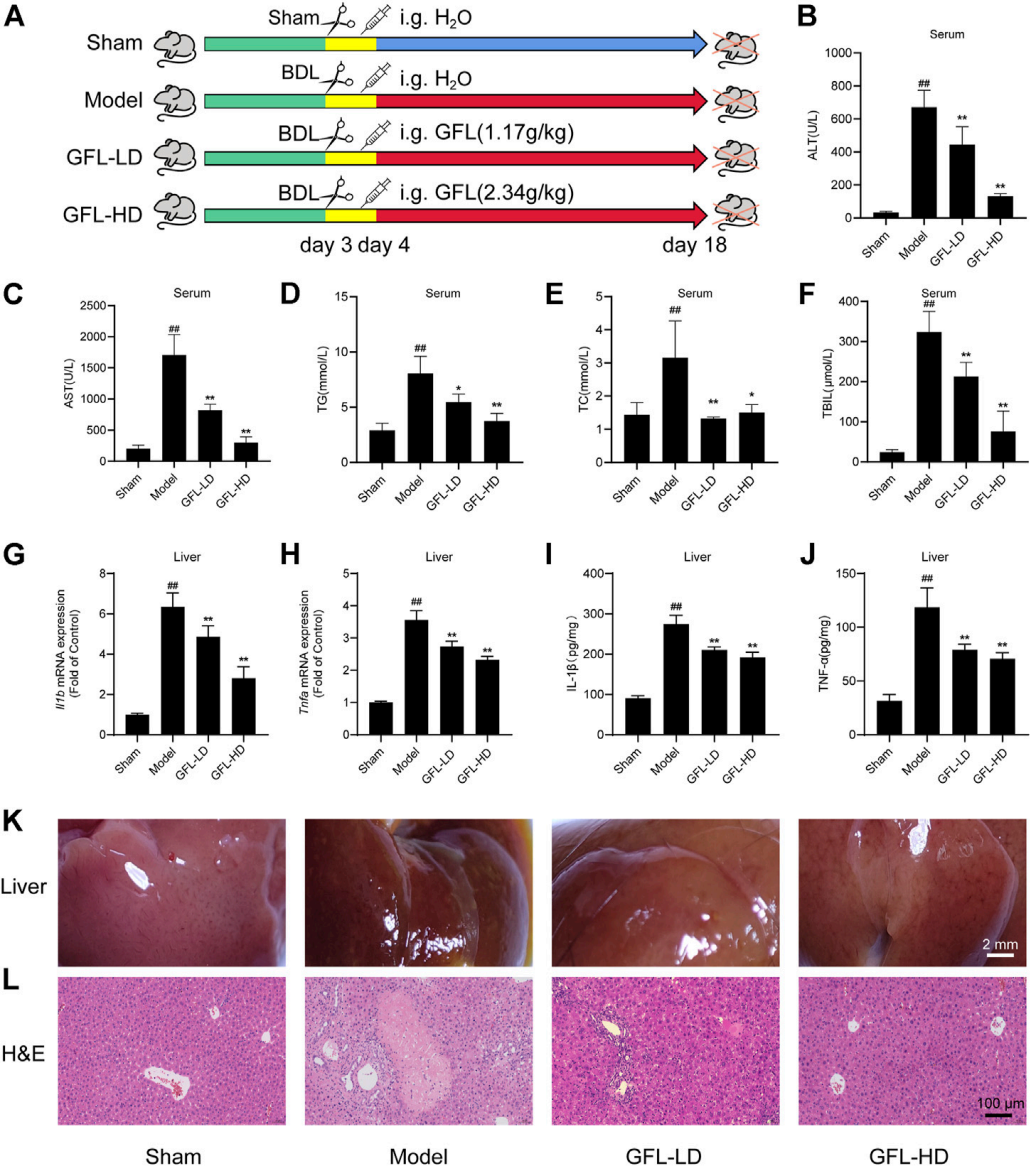
Effect of GFL on liver injury and inflammation in mice. **(A)** C57BL/6 mice were divided into sham (sham operation), model (bile duct ligation), GFL-LD (1.17 g/kg), and GFL-HD (2.34 g/kg) groups. In the sham group, the bile duct was separated but not ligated. In the model, GFL-LD, and GFL-HD groups, the bile duct was ligated. GFL-LD and GFL-HD groups were treated with different concentrations of GFL for 2 weeks 24 h after bile duct ligation. The Sham and model groups were given equal volume of normal saline. Contents of **(B)** AST, **(C)** ALT, **(D)** TG, **(E)** TC, and **(F)** TBIL in serum. mRNA expression of **(G)** IL-1β and **(H)** TNF-α in the liver. Protein expression of **(I)** IL-1β and **(J)** TNF-α in the liver. **(K)** Superficial view of liver tissue (×4 magnification). **(L)** Representative H&E staining images of liver tissue sections (×200 magnification).

A mice model of BDL-induced liver fibrosis was successfully established. The effect of GFL on BDL-induced liver fibrosis was evaluated by comparing GFL-treated groups to sham-operated and model groups (Figure 2A). As compared to the sham group, the results of serum analysis revealed a substantially higher level of ALT and AST in the model group, whereas the expressions of both liver injury-related enzymes were significantly reduced in GFL-treated groups in a dose-related manner (Figures 2B,C). Concurrently, abnormal levels of serum TG and TC indicated hyperlipidemia in the model group, while serum profiles of GFL-treated groups demonstrated a significant reduction in their TG and TC levels (Figures 2D,E). During the experiment, the mice in the model group and GFL-LD evidently had jaundice. Jaundice is usually caused by elevated levels of TBIL in the blood. Therefore, we detected the TBIL content in the serum and found that the TBIL level in the model was significantly higher than that in the sham-operated group. GFL could significantly restrict the rise of BDL-induced serum TBIL (Figure 2F) in a dose-dependent manner. Next, we detected the level of inflammation in the liver. The results showed that the expression of inflammatory factors IL-1β (*Il1b*), TNF-α (*Tnfa*), mRNA, and protein in the model group increased significantly, and GFL intervention could significantly inhibit the expression of inflammatory factors (Figures 2G–J).

The morphological examination of the liver tissues showed a significantly rougher surface and darkened red color of the liver of the mice in the model in comparison to the liver tissue of sham-operated mice. The surface of the liver of the model group also appeared slightly collapsed. On the other hand, the liver of GFL-treated mice presented a smoother surface with a brighter color than that of model mice, indicating an obvious effect of GFL (Figure 2K). The results of H&E staining implied a severely damaged histological structure of the liver in the model by showing extensive hepatic parenchymal necrosis, collagen deposition, inflammatory infiltration, and hyperplastic bile ducts. Conversely, these pathological changes were significantly minimized in the GFL-treatment group (Figure 2L). These results suggested that GFL can ameliorate BDL-induced liver injury and inflammation and may play a therapeutic role in liver fibrosis.

### 3.3 GFL can improve BDL-induced liver fibrosis in mice

**FIGURE 3:**
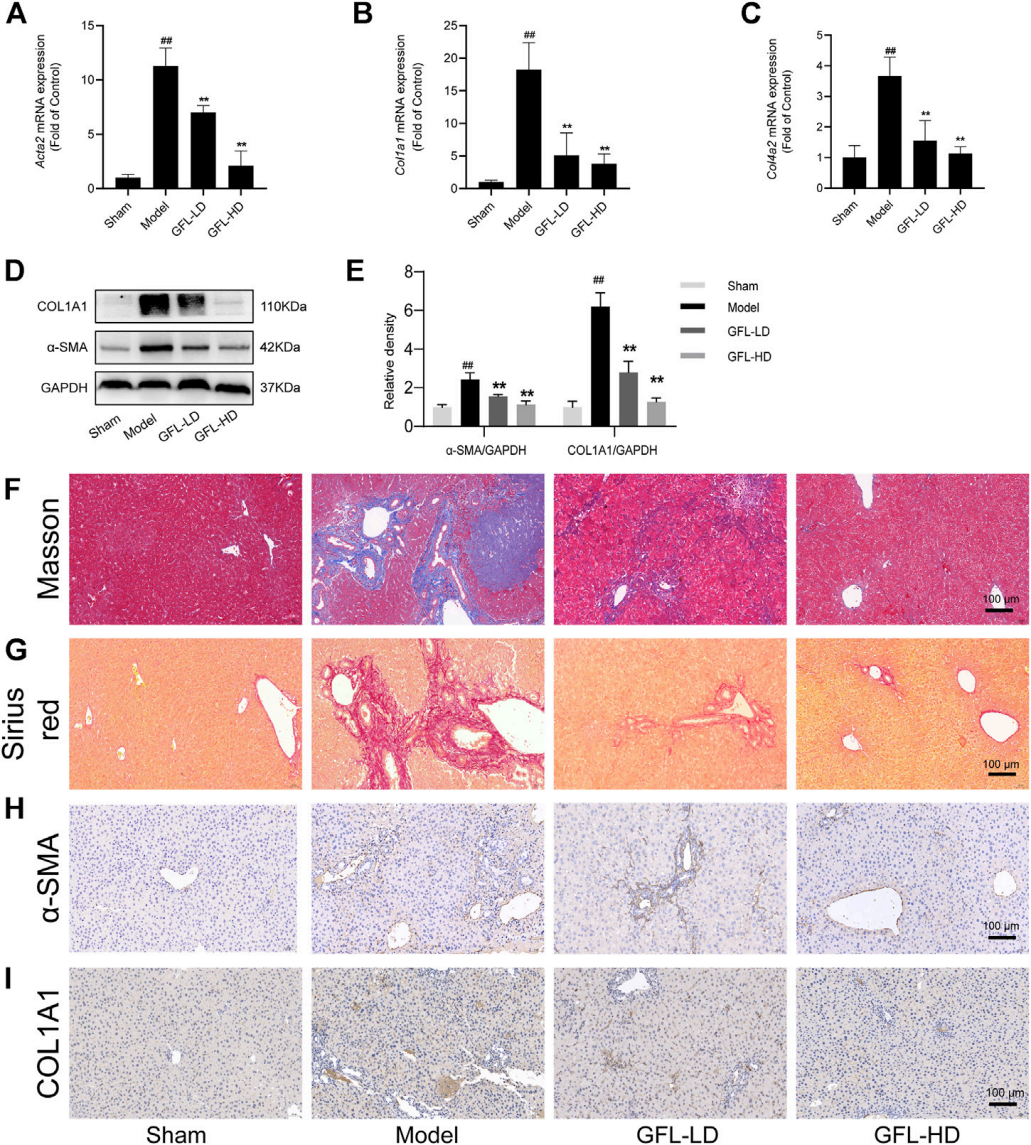
Effects of GFL on liver fibrosis in mice. mRNA expression of **(A)** α-SMA, **(B)** COL1A1, and **(C)** COL4A2 in the liver. **(D)** Protein expression of α-SMA and COL1A1 in the liver. **(E)** Protein expression statistics of α-SMA and COL1A1. **(F)** Representative MASSON-stained images of liver tissue sections (×200 magnification). **(G)** Representative Sirius red-stained images of liver tissue sections (×200 magnification). Representative **(H)** α-SMA and **(I)** COL1A1 immunohistochemical images of liver tissue sections (×200 magnification). Data are expressed as the mean ± SD, n = 6. Data were analyzed using one-way ANOVA. #*p* < 0.05, ##*p* < 0.01 compared to the sham group. *p < 0.05, **p < 0.01 compared to the model group.

We next evaluated the therapeutic potential of GFL against liver fibrosis in BDL mice. First of all, we detected the mRNA expression of collagen deposition genes COL1A1(*Col1a1*), COL4A2(*Col4a2*), and ECM proliferation-related gene α-SMA (*Acta2*) in liver tissue. We found that COL1A1, COL4A2, and α-SMA mRNA expressions were significantly increased in the liver of the model, while the transcription of such liver fibrosis-related genes was significantly inhibited by GFL (Figures 3A–C). Likewise, GFL inhibited the translation of COL1A1 and α-SMA at the protein level (Figures 3D,E). Furthermore, we used MASSON staining and Sirius red staining to detect collagen deposition in tissue sections to assess the extent of fibrosis. In the liver of mice in the model group, a large amount of collagen deposition appeared in the portal vein region, and this collagen deposition even spread to the liver parenchyma region. After GFL intervention, there was only a small amount of collagen deposition near the hepatic hilum without a notable collagen deposition in the liver parenchyma (Figures 3F,G). In addition, the immunohistochemical results of α-SMA and COL1A1 further proved the pharmacological effects of GFL in the treatment of liver fibrosis. The positive areas which were more prominent in the liver of mice in the model group decreased after GFL intervention (Figures 3H,I). The results showed that GFL could inhibit the activation of hepatic stellate cells. We proved it in *in vitro* experiments. GFL inhibited the activation of lx-2 cells stimulated by TGF-β1 (Supplementary Figure S1). In conclusion, GFL could effectively treat BDL-induced liver fibrosis in mice.

### 3.4 GFL alleviates BDL-induced serum metabolic disorder in mice

**FIGURE 4:**
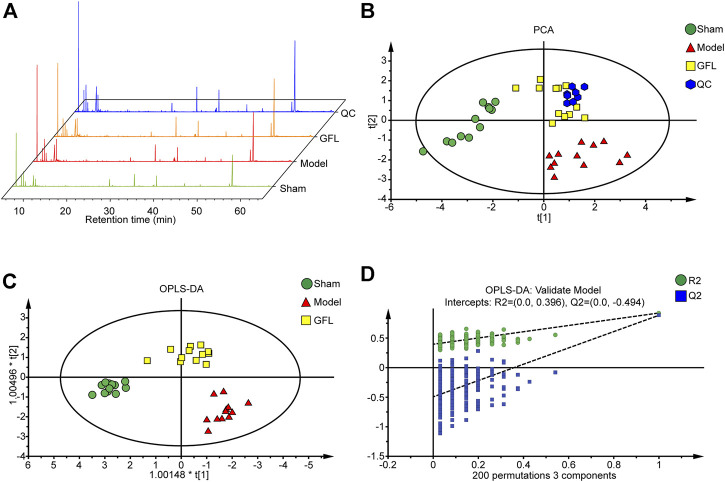
Metabolomic analysis of metabolic disorders in BDL mice by GFL. **(A)** TIC map of total ion currents of GC-MS metabolites in serum of mice in each group. **(B)** PCA analysis of serum GC-MS metabolites in mice in each group. **(C)** Analysis of serum GC-MS metabolites OPLS-DA of mice in each group. **(D)** In total, 200 permutation tests were performed on the OPLS-DA model.

Following the confirmation of the pharmacological effects of GFL in relieving liver injury and treating liver fibrosis, a metabolomic analysis was performed on the serum of mice from the sham, model, and GFL-HD groups. The GFL-HD group was selected due to better liver injury and fibrosis-alleviating effects. The total ion flow diagram showed that the metabolites in the model group changed significantly compared with the sham group, while the GFL group could callback some changes (Figure 4A). The principal component analysis (PCA) was then performed by processing the data of the sham, model, GFL, and QC in SIMCA-P (Figure 4B). The results showed a good aggregation in the PCA of the QC samples, indicating the reliability of the experimental and data processing methods. The samples of the sham and model were obviously separated in the PCA. The GFL was distributed between the sham-operated and model groups with an evident trend of recovery. The values of R2 (0.603) and Q2 (0.394) indicated a good quality of the PCA model, but its prediction ability required improvement. Therefore, we used orthogonal partial least squares (OPLS-DA) for further analysis (Figure 4C). In the OPLS-DA model, the sham group samples were well separated from the model, and the GFL group had a trend of recovery toward the sham. The R2X, R2Y, and Q2 were 0.688, 0.858, and 0.758, respectively, in the OPLS-DA model, indicating that this model had good quality and predictive ability. The overfitting of the OPLS-DA model was determined by external validation of 200 permutation tests, and the resulting R2 = 0.396, Q2 = −0.494, R2 < 0.4, and Q2 < 0 revealed that the OPLS-DA model was not overfitting (Figure 4D). The aforementioned results show that after BDL surgery in mice, the endogenous metabolites were disordered. However, these metabolic abnormalities could be successfully reversed by treatment with GFL.

### 3.5 GFL affects glutamine metabolism to relieve liver fibrosis

**FIGURE 5:**
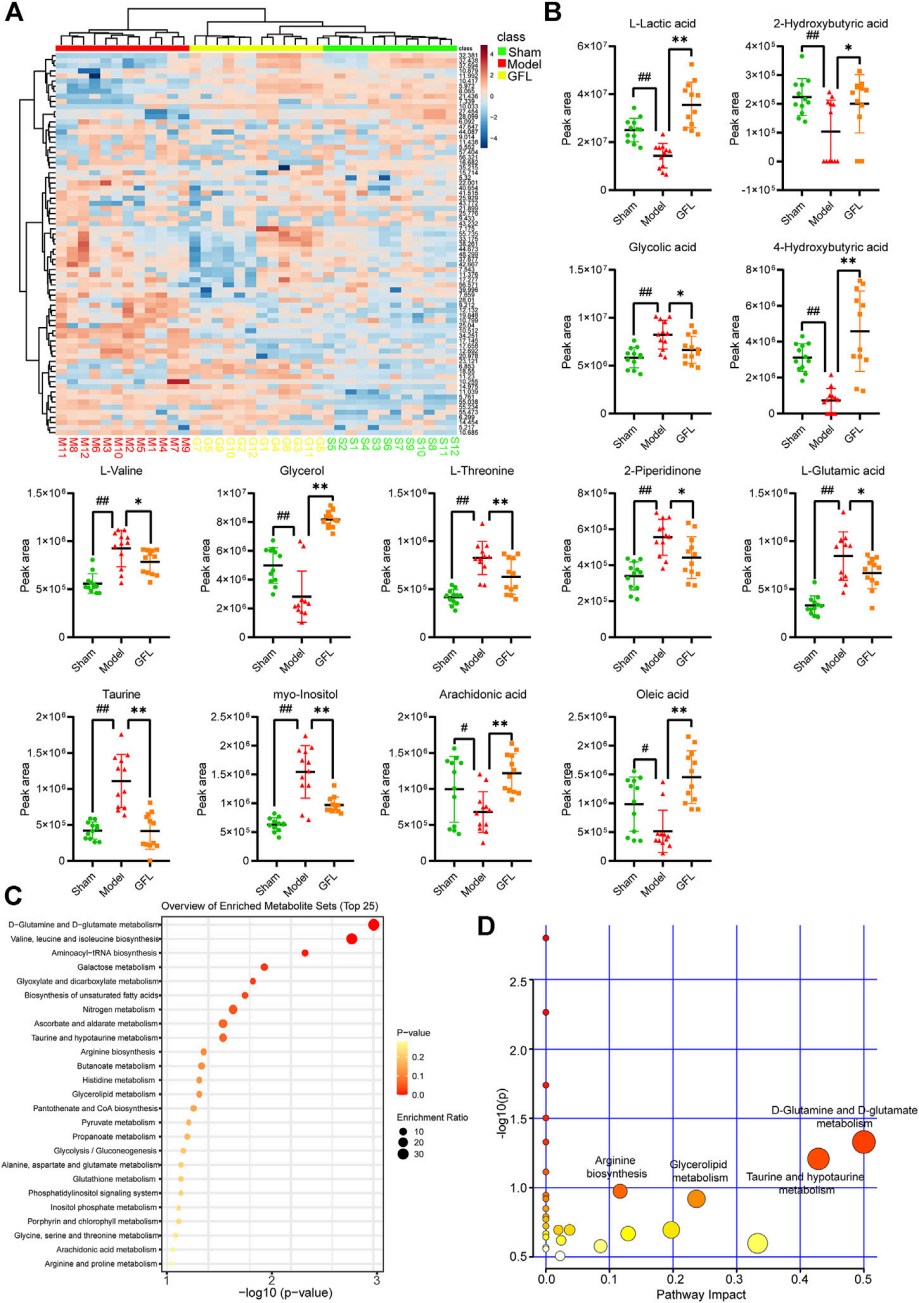
Data analysis of GC-MS endogenous metabolites. **(A)** Heat map of serum metabolic related to hepatic fibrosis induced by GFL in each group of mice. Color represents the concentration of metabolites, from low (blue) to high (red). Green represents a fake surgical group, red represents the model group, and yellow represents the GFL group. **(B)** Peak area of differential metabolites. **(C)** Enrichment analysis of differential metabolites. **(D)** Pathways analysis of differential metabolites. Data are expressed as the mean ± SD, n = 12. Data were analyzed using one-way ANOVA. ^#^ *p* < 0.05, ^##^ *p* < 0.01 compared to the sham group. *p < 0.05, **p < 0.01 compared to the model group.

A cluster analysis was performed following the normalization of the values representing the sample metabolites of each group, and the results are shown in a heat map (Figure 5A) with each region representing the relative concentration of a metabolite in each set of the samples. The compounds with VIP value > 1 and significant difference between groups (*p* <0.05) in the OPLS-DA model were selected. The peaks that met the two conditions at the same time were considered differential metabolites. These compounds are identified and presented in Table 2. Compared with the sham-operated group, the concentration of six metabolites in the model group decreased substantially but increased significantly following GFL administration. On the other hand, the concentration of seven metabolites in the model increased substantially, followed by a significant downregulation after GFL administration. These compounds are l-lactic acid, 2-hydroxybutyric acid, glycolic acid, 4-hydroxybutyric acid, l-valine, glycerol, l-threonine, 2-piperidinone, l-glutamic acid, taurine, myo-inositol, arachidonic acid, and oleic acid. The GC-MS peak areas of these compounds are shown in Figure 5B. For these compounds, their enrichment analysis was performed using MetaboAnalyst 6.0. The results showed that the top-ranked pathways were d-glutamine and d-glutamate metabolism; valine, leucine, and isoleucine biosynthesis; aminoacyl-tRNA biosynthesis; and other pathways (Figure 5C). These enrichment analyses suggest that GFL could alleviate metabolic disorders and treat liver fibrosis by affecting multiple metabolic pathways. Similarly, a pathway analysis of these compounds (Figure 5D) underlined d-glutamine and d-glutamate metabolism as the most significant. The metabolism of taurine, hypotaurine, and glycerolipid and biosynthesis of arginine were ranked before the bubble map at −log10(p) > 0.75 with pathway impact > 0.1. Based on the aforementioned results, the remission of liver fibrosis by GFL may be related to glutamine metabolism.

**TABLE 2:** Differential metabolites.

No.	Retention time (min)	Metabolite	Molecular formula	Molecular weight	VIP	Model vs. sham		GFL vs. model	
						Trend	P	Trend	P
1	5.97	l-Lactic acid	C3H6O3	90.078	1.30585	↓	0.00001	↑	0.00001
2	7.34	2-Hydroxybutyric acid	C4H8O3	104.105	1.04449	↓	0.00546	↑	0.03442
3	7.66	Glycolic acid	C2H4O3	76.051	1.24028	↑	0.00009	↓	0.00739
4	8.07	4-Hydroxybutyric acid	C4H8O3	104.105	1.25554	↓	0.00001	↑	0.00001
5	9.21	l-Valine	C5H11NO2	117.146	1.01277	↑	0.00001	↓	0.02185
6	10.42	Glycerol	C3H8O3	92.094	1.4135	↓	0.00109	↑	0.00001
7	12.69	l-Threonine	C4H9NO3	119.119	1.09762	↑	0.00001	↓	0.00648
8	17.66	2-Piperidinone	C5H9NO	99.1311	1.05744	↑	0.00001	↓	0.00876
9	19.85	l-Glutamic acid	C5H9NO4	147.129	1.09292	↑	0.00001	↓	0.02597
10	20.98	Taurine	C2H7NO3S	125.147	1.3687	↑	0.00001	↓	0.00001
11	34.25	Myo-inositol	C6H12O6	180.156	1.21654	↑	0.00001	↓	0.00019
12	37.44	Arachidonic acid	C20H32O2	304.467	1.0715	↓	0.02666	↑	0.00004
13	37.59	Oleic acid	C18H34O2	282.461	1.21582	↓	0.00610	↑	0.00001

### 3.6 GFL inhibits IκB-α/NF-κB phosphorylation to adjust glutaminase activity

**FIGURE 6:**
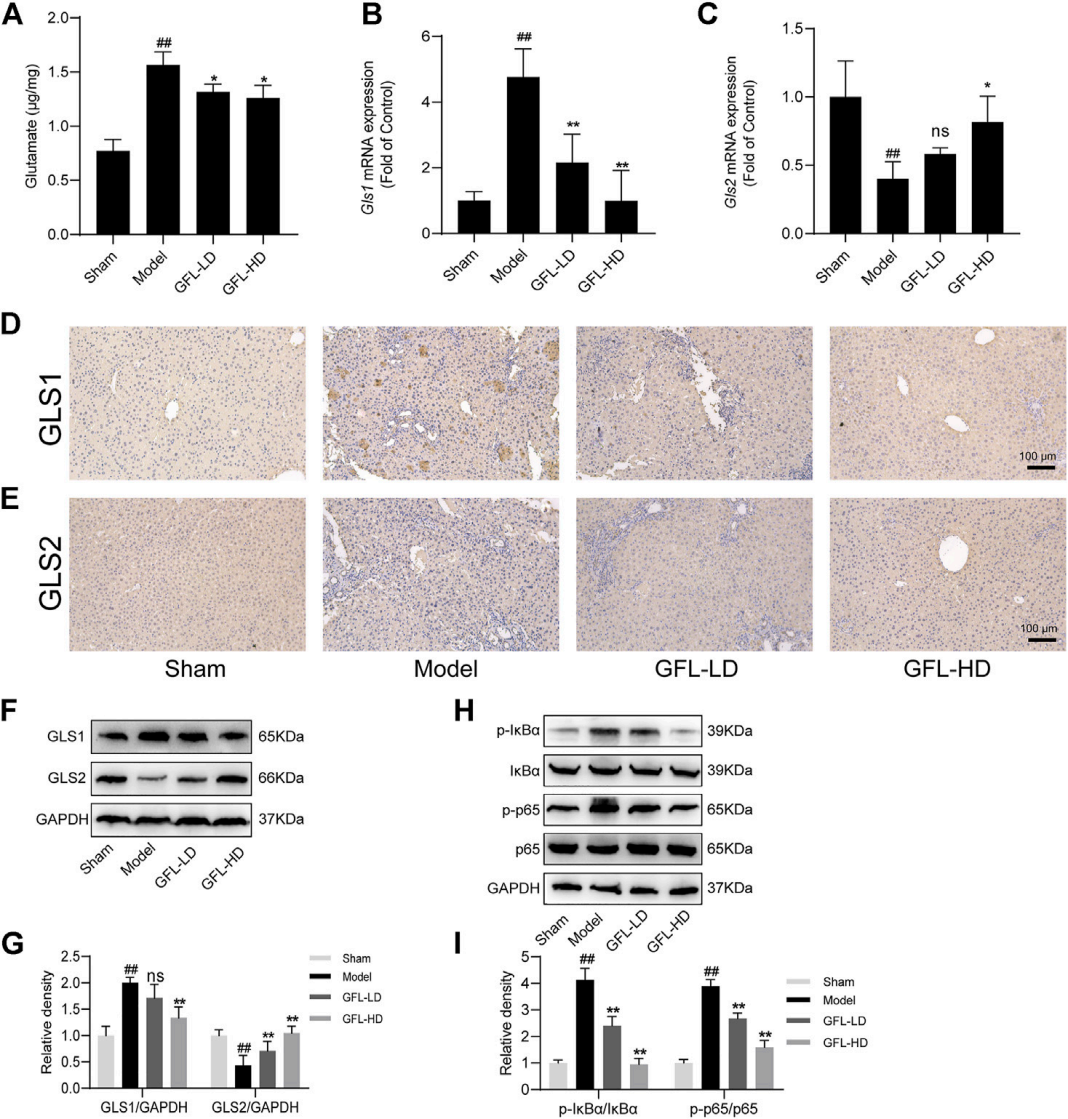
GFL regulation of glutamine metabolism by the NF-κB pathway. **(A)** Glutamate content in the liver. mRNA expression of **(B)** GLS1 and **(C)** GLS2 in the liver. Representative **(D)** GLS1 and **(E)** GLS2 immunohistochemical images of liver tissue sections (×200 magnification). **(F)** Protein expression of GLS1 and GLS2 in the liver. **(G)** Protein expression statistics of GLS1 and GLS2. **(H)** Protein expression of the NF-κB pathway in the liver. **(I)** Protein expression statistics of the NF-κB pathway. Data are expressed as the mean ± SD, n = 3. Data were analyzed using one-way ANOVA. #*p* < 0.05, ##*p* < 0.01 compared to the sham group. *p < 0.05, **p < 0.01 compared to the model group.

**FIGURE 7:**
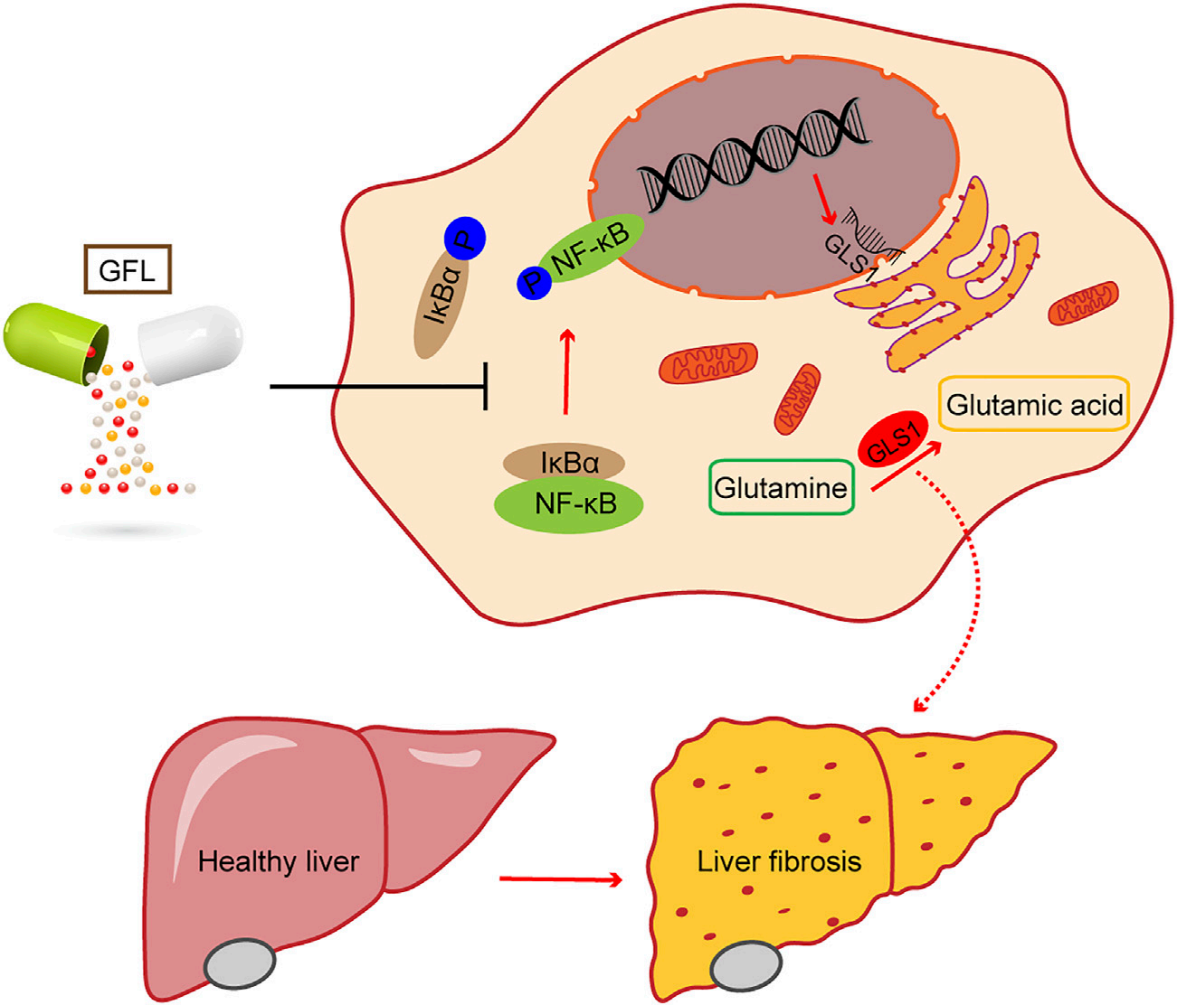
Phosphorylation of the NF-κB pathway regulates GLS1 transcription and translation, while GFL can inhibit the activation of the NF-κB pathway, reduce GLS1 expression, regulate glutamine metabolism, alleviate liver injury, and relieve liver fibrosis.

An increase in glutamate in the liver has been associated with disorders of glutamine metabolism, which occur during the course of liver fibrosis (Choi et al., 2021; Wang et al., 2021). Glutamic acid is the metabolic product of glutamine. Therefore, we assessed the content of glutamic acid to estimate glutamine metabolic disorders.3 The results showed that the content of glutamate in the liver of model mice was significantly higher than that of the control, but it decreased after GFL intervention (Figure 6A). Furthermore, we detected the expression of the glutamine metabolic enzyme GLS which mainly has two subtypes: kidney GLS1 and hepatic GLS2. We proved through a variety of means that GLS1 had a significant increasing trend after the transcription and translation of the model and the expression of GLS1, and this trend could be inhibited by GFL treatment whether in terms of mRNA level or protein level (Figures 6B,D,F). Conversely, the expression of GLS2 in the model mice had decreasing trend which could be inhibited by drug intervention as well (Figures 6C,E,F). As NF-κB is activated in almost all chronic liver diseases and can regulate glutaminase expression (Wang et al., 2010), we detected the expression of NF-κB pathway-related proteins. The results showed uplifted NF-κB p65 and the kinase IκB-α phosphorylation in the liver of the BDL mice. Furthermore, GFL intervention could also inhibit NF-κB pathway protein phosphorylation levels. These results suggest that GFL may regulate glutamine metabolism by inhibiting the NF-κB pathway (Figure 7).

## 4 Discussion

The present study proved the potential of GFL to inhibit BDL-induced liver injury by alleviating the levels of ALT, AST, TC, TG, and TBIL in serum. Serum ALT and AST levels are important and sensitive biochemical indicators for assessing liver functions (Cao et al., 2015). Because the liver is the main organ of blood lipid metabolism, TC and TG are also increased during liver injury (Bechmann et al., 2012). TBIL is the main substance of the phenomenon of jaundice (Hamoud et al., 2018) and serves as a common indicator for clinical detection of hepatic injury. In the present study, jaundice was strongly indicated in BDL-receiving mice during feeding, and it was mitigated or completely treated after GFL administration. Chronic inflammatory infiltration is one of the key factors of liver fibrosis (Calvente et al., 2019). We detected the transcription and translation of inflammatory factors such as IL-1β and TNF-α in the liver, and proved the inhibitory effect of GFL on BDL-induced hepatitis. The pathological morphology of the liver is a gold standard for liver disease detection. The American Association for the Study of Liver Diseases (AASLD) recommends liver biopsy in NAFLD patients who are at a high risk of advanced liver fibrosis. Under direct observation and H&E dyeing on the liver, bile duct-ligated mice presented obvious liver injury, rough liver surface, intrahepatic bile duct dilatation, and apparent collagen deposition. The GFL treatment diminished these pathological changes resulting in a smooth surface and lowered bile duct dilatation. These results confirmed the therapeutic effect of GFL on BDL-induced liver injury.

BDL causes extrahepatic biliary obstruction and thereby results in bile duct dilatation and cholestasis. Subsequently, the intrahepatic blood vessels are compressed causing bile extravasation. The consequent suffering of hepatocytes from ischemia and necrosis initiates the process of fibrosis (Ghallab et al., 2019). The essence of liver fibrosis is that HSCs are activated and turned into myofibroblasts resulting in ECM proliferation and collagen deposition. Since the α-SMA is considered a marker of HSCs (Puche et al., 2013) and COL1A1 is regarded as the main type of collagen deposition in ECM (Kular et al., 2014), we used RT-PCR, Western blot, and IHC technology to highlight the mRNA and protein levels of α-SMA and COL1A1 in the liver. After experiencing BDL, the transcription and translation of liver tissue α-SMA increased significantly and collagen1 was deposited. The model exhibited severe liver fibrosis, while GFL treatment significantly decreased α-SMA and lightened collagen deposition, which were consistent with our findings in LX-2 cells. Furthermore, Masson staining and Sirius red staining were used for detecting the distribution of collagen in liver paraffin sections due to their capability of directly showing the deposition of collagen. The two staining results clearly supported the view that GFL can alleviate hepatic fibrosis in bile duct-ligated mice.

The importance of the liver as a metabolic center of the body signifies the close association between the development of liver fibrosis and metabolism. Liver injury can lead to drastic changes in intrahepatic metabolism. The demand for energy increases in the process of liver fibrosis due to the extensive proliferation of fibroblasts and simultaneous supply of other components to the damaged area such as ECM, proteas, and cytokines. To meet these energy needs, cells use many metabolic pathways similar to cancer cells (Lane et al., 2020). We, therefore, analyzed the liver samples by GC-MS and enriched the metabolites that could be affected by GFL. The results implied that GFL may alleviate liver injury and inhibit liver fibrosis by affecting d-glutamine and d-glutamate metabolism; taurine and hypotaurine metabolism; glycerolipid metabolism; and biosynthesis of arginine. The taurine pathway plays an antioxidant role in acute liver failure (Mizota et al., 2022), and it can inhibit liver injury and liver fibrosis by inhibiting TLR4/NFKB and (Younis et al., 2021) and TxNiP/NLRP3 (Yao et al., 2021) pathways. Because arginine can inhibit CCL4-induced liver fibrosis in mice (Leung et al., 2011), inhibition of arginine synthesis would ultimately lead to hepatic fibrosis (Wu et al., 2019). Regarding phospholipase and TG hydrolase, their activity would affect the level of liver LCA-COA and TG, which cause chronic biliary sludge-mediated liver injury (Moustafa et al., 2012). With reference to glutamine, its decomposition enhances the translation and stability of collagen and subsequently increases the expression of XIAP and survivin, which are members of the apoptotic protein (IAP) family. The end products of α-ketoglutarate (α-Kg) decomposition could enhance the anti-apoptotic ability of myofibroblasts.

The metabolic analysis in the current study found that glutamine metabolism topped among all studied metabolic pathways. We chose the glutamine metabolic pathway for further research because of its close association with liver fibrosis. The activation of HSCs causes liver fibrosis, which has a strong energy demand, while glutamine decomposition (where glutamine is converted into α-ketoglutarate) has been proved to provide energy for the activation of HSCs; thus, inhibition of glutamine metabolism may be a possible target for the treatment of liver fibrosis (Du et al., 2018). GLS is the main rate-limiting enzyme of glutamine metabolism, including GLS1 and GLS2 subtypes. As compared with GLS2, GLS1 is the main contributor to cellular glutaminase activity and plays a leading role in glutamine metabolism (Wang et al., 2021). The inhibition of its activity could significantly minimize the production of experimental fibrosis (Ge et al., 2018; Bai et al., 2019; Cui et al., 2019). Meanwhile, it has been shown that glutamine metabolism in the liver transforms from low-activity GLS2 subtypes to high-activity GLS1 subtypes at the end of chronic liver disease (Xiang et al., 2015; Yu et al., 2015; Du et al., 2018). Concurrently, as the raw material of glutathione synthesis, the excessive decomposition of glutamine will inhibit the synthesis of glutathione and accelerate the depletion of glutathione, which will undoubtedly promote the generation of liver fibrosis (Perina et al., 2019). Through different methodological approaches, we found higher transcription and translation of GLS1 in non-GFL-treated BDL-induced mice than that in GFL-treated BDL-induced mice. On the other hand, the expression of GLS2 decreased in non-interventional bile duct-ligated mice, but its level in interventional mice returned to that seen in the sham group. Similarly, the content of glutamate (glutamine metabolite) in the livers of mice groups demonstrated its GFL-mediated reversal. These results prove the effectiveness of GFL in regulating the metabolic abnormalities in BDL mice, especially glutamine metabolism.

NF-κB controls the transcriptions of hundreds of genes, and plays an essential role in inflammation, immunity, cell proliferation, differentiation, and survival (Oeckinghaus et al., 2011). Its activation has been reported in most chronic liver diseases, including alcoholic liver disease, NAFLD, viral hepatitis, and biliary liver disease (Luedde and Schwabe, 2011). The NF-κB-related activity of glutaminase can be inhibited by upstreaming kinase IKKβ or the p65/RELA subunit that interferes with the activity of NF-κB at different levels (Sacks et al., 2018). Our study proved that GFL can inhibit glutamine metabolism by restricting the activation of the NF-κB pathway. As other NF-κB-associated targets are also being identified for liver diseases, such as Rho GTPases (Zhang et al., 2018), whether GFL directly acts on the NF-κB pathway, thereby affecting glutamine metabolism, requires further research. Using knockout mice or cell lines to more clearly demonstrate the mechanism of GFL, regulating glutamine metabolism and inhibiting liver fibrosis may be necessary.

GFL is composed of Codonopsis radix, Trionycis Carapax, Paridis Rhizoma, Atractylodis Macrocephalae Rhizoma, Astragali Radix, etc*.* Its ability to treat liver cancer has been confirmed (Xu et al., 2022). Many studies have shown that the components of GFL have evident pharmacological effects on liver fibrosis. The extract of Codonopsis radix can regulate the expression of matrix metalloproteinases in rats with liver fibrosis induced by hepatectomy, promote liver regeneration, and inhibit liver fibrosis (Wu et al., 2015). Flavonoids in Astragali Radix have also been proved to inhibit the activation of HSCs by inhibiting the NF-κB pathway (Sun et al., 2012), and turtle shell (Trionycis Carapax) decoction can block the TGF-β–SMAD pathway inhibiting liver fibrosis (Bai et al., 2016). However, the effect and mechanism of GFL on liver fibrosis is unclear. The previous studies on the treatment of liver fibrosis with GFL mainly focused on clinical studies and pharmacodynamic studies, and there was little discussion on the mechanism of GFL (M Wang and T Liu, 2016; Chen et al., 2000). Our research is an effective supplement to whether GFL, which is composed of a variety of medicinal materials, has notable pharmacological effects on liver fibrosis and what is the possible mechanism.

## 5 Conclusion

Our study shows that GFL can inhibit glutamine metabolism which was correlated with the NF-κB pathway, so as to restore the effect of alleviating liver fibrosis. To some extent, this study fills the gap in the mechanism of GFL and provides a reference for the clinical use of GFL in the treatment of liver fibrosis.

## Data Availability

The original contributions presented in the study are included in the article/Supplementary Material; further inquiries can be directed to the corresponding authors.
