# Supplementary material for: Ganfule capsule alleviates bile duct ligation-induced liver fibrosis in mice by inhibiting glutamine metabolism
Source: Front Pharmacol. 2022 Oct 7;13:930785. doi: 10.3389/fphar.2022.930785 (PMC9585157; doi:10.3389/fphar.2022.930785)
Supplement: Supplementary file 2 [file Table1.docx]

| **Table S1** RT-PCR primer sequences | |
| --- | --- |
| Name | Sequence(5'-3') |
| Mus-β-actin |  |
| Forward | TGCTGTCCCTGTATGCCTCT |
| Reverse | TTTGATGTCACGCACGATTT |
| Mus-α-SMA |  |
| Forward | GTACCCAGGCATTGCTGACA |
| Reverse | GCTGGAAGGTAGACAGCGAA |
| Mus-COL1A1 |  |
| Forward | TTCTCCTGGCAAAGACGGAC |
| Reverse | CTCAAGGTCACGGTCACGAA |
| Mus-COL4A2 |  |
| Forward | CTGGTGAAGCACAGCCAAAC |
| Reverse | GTCACCCGGATTGCAGTACA |
| Mus-IL-1β |  |
| Forward | CTTCAGGCAGGCAGTATCACTC |
| Reverse | TGCAGTTGTCTAATGGGAACGT |
| Mus-TNF-α |  |
| Forward | CCTCTAGCCCACGTCGTAGC |
| Reverse | AGCAATGACTCCAAAGTAGACC |
| Mus-GLS1 |  |
| Forward | CATCCTCATCTGACGAGCGG |
| Reverse | TCCTGTAGGATCTCCGAGGG |
| Mus-GLS2 |  |
| Forward | GGATCATGACGCCTCACACA |
| Reverse | TGCTGCTCACACACTTTTGG |
| Human-β-actin |  |
| Forward | GACAGTCAGCCGCATCTTCT |
| Reverse | GCGCCCAATACGACCAAATC |
| Human-α-SMA |  |
| Forward | CCTATCCCCGGGACTAAGAC |
| Reverse | CCATCACCCCCTGATGTCTG |

| Human-COL1A1 |  |
| --- | --- |
| Forward | AAGCTGGAAAACCTGGTCGT |
| Reverse | AGCACCATCATTTCCACGAG |
| Human-COL4A2 |  |
| Forward | GGACAGACGAGACAACAGCA |
| Reverse | GAGCTGGCATAACATTGGCG |
