# Supplementary material for: Ganfule capsule alleviates bile duct ligation-induced liver fibrosis in mice by inhibiting glutamine metabolism
Source: Front Pharmacol. 2022 Oct 7;13:930785. doi: 10.3389/fphar.2022.930785 (PMC9585157; doi:10.3389/fphar.2022.930785)
Supplement: Supplementary file 3 [file Image1.pdf]

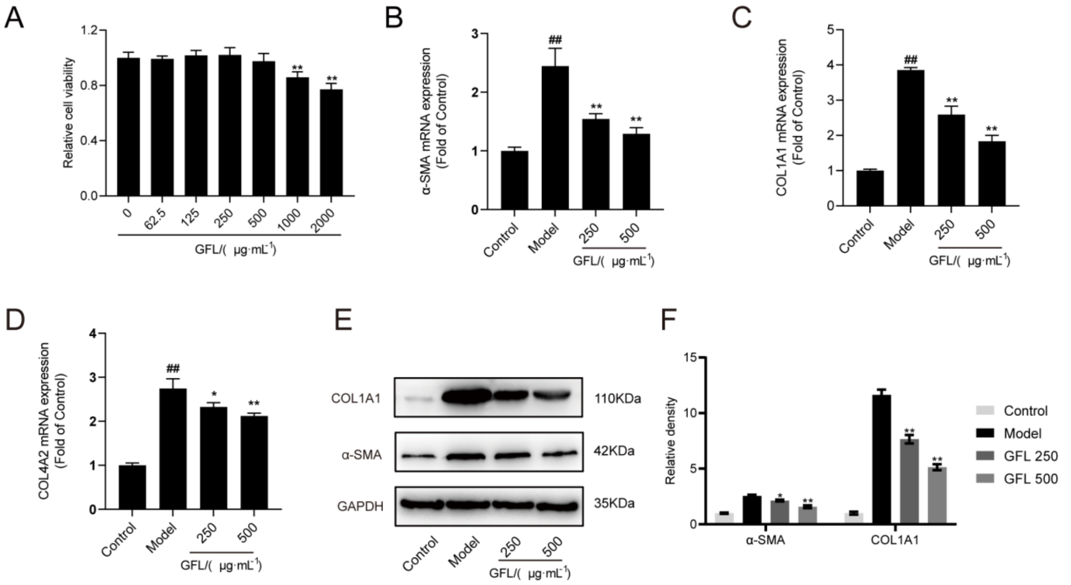

Fig. S1. GFL inhibits TGF-β1 stimulated LX-2 activation . (A) Effects of different concentrations of GFL on the viability of LX-2, mRNA expression of (B) α-SMA, (C) COL1A1, and (D) COL4A2 in LX-2. (E) Protein expression of α-SMA and COL1A1 in LX-2. (F) Protein expression statistics of α-SMA and COL1A1. Data are expressed as the mean ± SD, n=3. Data were analyzed using one-way ANOVA. #p < 0.05, ##p < 0.01 compared to the sham group. *p < 0.05, **p < 0.01 compared to the model group.
